# Plasma multi-omics reveals molecular remodeling and prognostic biomarkers in advanced breast cancer treated with trastuzumab deruxtecan

**DOI:** 10.3389/fonc.2026.1973097

**Published:** 2026-09-18

**Authors:** Die Sang, Wenyu Yang, Jinsong Liu, Yumeng Li, Shanshan Zhao, Xue Wang, Yurong Zhang, Biyun Wang, Man Li, Peng Yuan

**Affiliations:** 1 Department of Medical Oncology, Beijing Chaoyang District Sanhuan Cancer Hospital, Beijing, China; 2 National Cancer Center/National Clinical Research Center for Cancer/Cancer Hospital, Chinese Academy of Medical Sciences and Peking Union Medical College, Beijing, China; 3 Department of VIP Medical Services, National Cancer Center/National Clinical Research Center for Cancer/Cancer Hospital, Chinese Academy of Medical Sciences and Peking Union Medical College, Beijing, China; 4 Department of Medical Oncology, Fudan University Affiliated Cancer Hospital, Shanghai, China; 5 Department of Oncology, The Second Hospital of Dalian Medical University, Dalian, China

**Keywords:** advanced breast cancer, biomarkers, metabolomics, multi−omics integration, proteomics, trastuzumab deruxtecan

## Abstract

**Background:**

Trastuzumab deruxtecan (T-DXd), a HER2-targeted antibody-drug conjugate, has demonstrated clinical activity in HER2-positive and HER2-low advanced breast cancer. However, treatment outcomes remain heterogeneous, and circulating molecular correlates of treatment and prognosis are incompletely defined.

**Methods:**

Twenty-nine patients receiving T-DXd were included in the clinical cohort, of whom 16 provided paired pre- and post-two-cycle plasma for multi-omics profiling. Proteomics used 4D-DIA LC-MS/MS; metabolomics used UHPLC-high-resolution MS in positive/negative modes. Treatment-associated features were identified by paired statistics, fold change, and multivariate criteria, followed by enrichment, correlation, joint pathway mapping, and exploratory PFS-oriented Cox/LASSO-Cox analyses.

**Results:**

Proteomics identified 4,444 proteins; 508 were differentially expressed (136 up, 372 down), enriched in translation/ribosome, complement/coagulation, acute-phase, cytoskeletal/adhesion, and lipid transport pathways. Metabolomics detected 3,596 features; 70 were differential (43 up, 27 down), enriched in amino acid, tryptophan, nucleotide/energy, and lipid metabolism. Cross-omics analysis revealed structured correlations and joint purine metabolism mapping. Exploratory baseline analyses selected six protein and four metabolite prognostic biomarkers for PFS visualization.

**Conclusion:**

Paired plasma multi-omics revealed T-DXd-associated proteomic and metabolic remodeling. The molecular patterns and PFS-associated biomarkers are hypothesis-generating and require validation in larger independent cohorts.

## 1 Introduction

HER2-positive breast cancer, defined by HER2 gene amplification or protein overexpression (IHC 3+ or IHC 2+/FISH-positive), accounts for 15–20% of all breast cancer cases (1). Although patients with HER2-positive advanced breast cancer initially respond well to HER2-targeted agents such as trastuzumab, the majority eventually develop resistance and experience disease progression (2). Moreover, a substantial proportion of breast cancers exhibit HER2-low expression (IHC 1+ or IHC 2+/FISH-negative, collectively designated HER2-low); historically classified as HER2-negative, these patients have lacked effective HER2-directed therapeutic options (3).

Trastuzumab deruxtecan (T-DXd) is a novel HER2-targeted ADC comprising a trastuzumab antibody, a cleavable tetrapeptide linker, and a topoisomerase I inhibitor payload, with a drug-to-antibody ratio of approximately 8 (4). Its mechanism of action encompasses antibody-mediated, site-specific delivery of cytotoxic payload to HER2-expressing tumor cells, followed by lysosomal linker cleavage, intracellular drug release, DNA damage, and apoptosis. Critically, the membrane-permeable payload exerts a “bystander effect” on adjacent HER2-low or HER2-negative tumor cells (5). Based on the landmark DESTINY-Breast04 trial, T-DXd has been approved for the treatment of HER2-low advanced breast cancer (6).

Despite its impressive clinical efficacy, a substantial subset of patients experience disease progression during T-DXd therapy. Established mechanisms of resistance include HER2 expression loss-approximately 49% of patients exhibit marked HER2 downregulation at progression, with complete HER2 loss occurring in half of these cases (7). HER2 extracellular domain mutations (e.g., p.V597M and p.P593R) that impair trastuzumab binding have also been reported to confer T-DXd resistance (8). Additional mechanisms encompass ABC transporter-mediated payload efflux and activation of alternative survival pathways. Accordingly, there is a pressing need to identify reliable biomarkers capable of predicting which patients will derive the greatest benefit from T-DXd and to elucidate the molecular underpinnings of drug action and resistance.

Multi-omics technologies offer complementary tools for addressing these challenges. Conventional HER2 assessment by immunohistochemistry (IHC) and fluorescence *in situ* hybridization (FISH) has inherent limitations for patient stratification, particularly in the HER2-low setting (9). Plasma proteomics enables interrogation of circulating proteins secreted or shed by tumor and host tissues, whereas metabolomics captures downstream functional readouts of cellular processes (10). Integrated proteomic and metabolomic analyses may therefore provide a broader view of the molecular effects of T-DXd, support the identification of candidate biomarkers (11), and generate hypotheses about drug action and resistance (12).The circulating molecular consequences of T-DXd exposure remain undescribed. Plasma enables repeated non-invasive sampling and captures systemic biology beyond tissue biopsies. We therefore adopted a paired multi-omics design integrating proteomics and metabolomics to close this gap and assess whether protein- and metabolite-level changes are coordinated and carry prognostic information.

In the present study, we profiled paired plasma proteomes and metabolomes before and after T-DXd treatment. We aimed to characterize treatment-associated molecular changes, identify the principal functional patterns represented in the datasets, and generate exploratory baseline prognostic biomarkers associated with PFS.

## 2 Materials and methods

### 2.1 Study design

Written informed consent was obtained from every patient prior to initiation of therapy, covering both the pre-treatment and post-treatment blood collections. The study protocol was reviewed and approved by the Ethics Committee of Beijing Chaoyang District Sanhuan Cancer Hospital (Approval No.:SH-2023034). A total of 114 patients with advanced breast cancer receiving T-DXd between March 2023 and December 2024 were initially screened. First, a clinical cohort of 29 patients was assembled from those with adequate clinical documentation and follow-up for outcome analyses; eligibility required a confirmed diagnosis of advanced or metastatic breast cancer, receipt of at least two cycles of T-DXd, and evaluable follow-up for progression-free survival. The principal reasons for exclusion were absence of an archived plasma sample at either time point or an unmatched pre-/post-treatment pair, withdrawal or discontinuation of T-DXd before completion of two cycles, insufficient sample volume or failure of sample quality control, and incomplete clinical or follow-up records; the corresponding numbers at each step are detailed in the study flow diagram (**Supplementary Figure S1**). Second, a paired multi-omics cohort of 16 patients was nested within this clinical cohort and comprised those who additionally had both pre-treatment and post-treatment (after 2 cycles of T-DXd) plasma samples available and passing quality control. All molecular analyses reported in this study, including the differential-expression, enrichment, cross-omics and baseline prognostic analyses, were performed exclusively in the 16-patient paired multi-omics cohort, which is therefore the primary analysis population. Peripheral blood was collected in EDTA tubes, immediately centrifuged to isolate plasma, and stored at −80 °C until analysis. The overall study workflow is illustrated in **Figure 1**.

**Figure 1:**
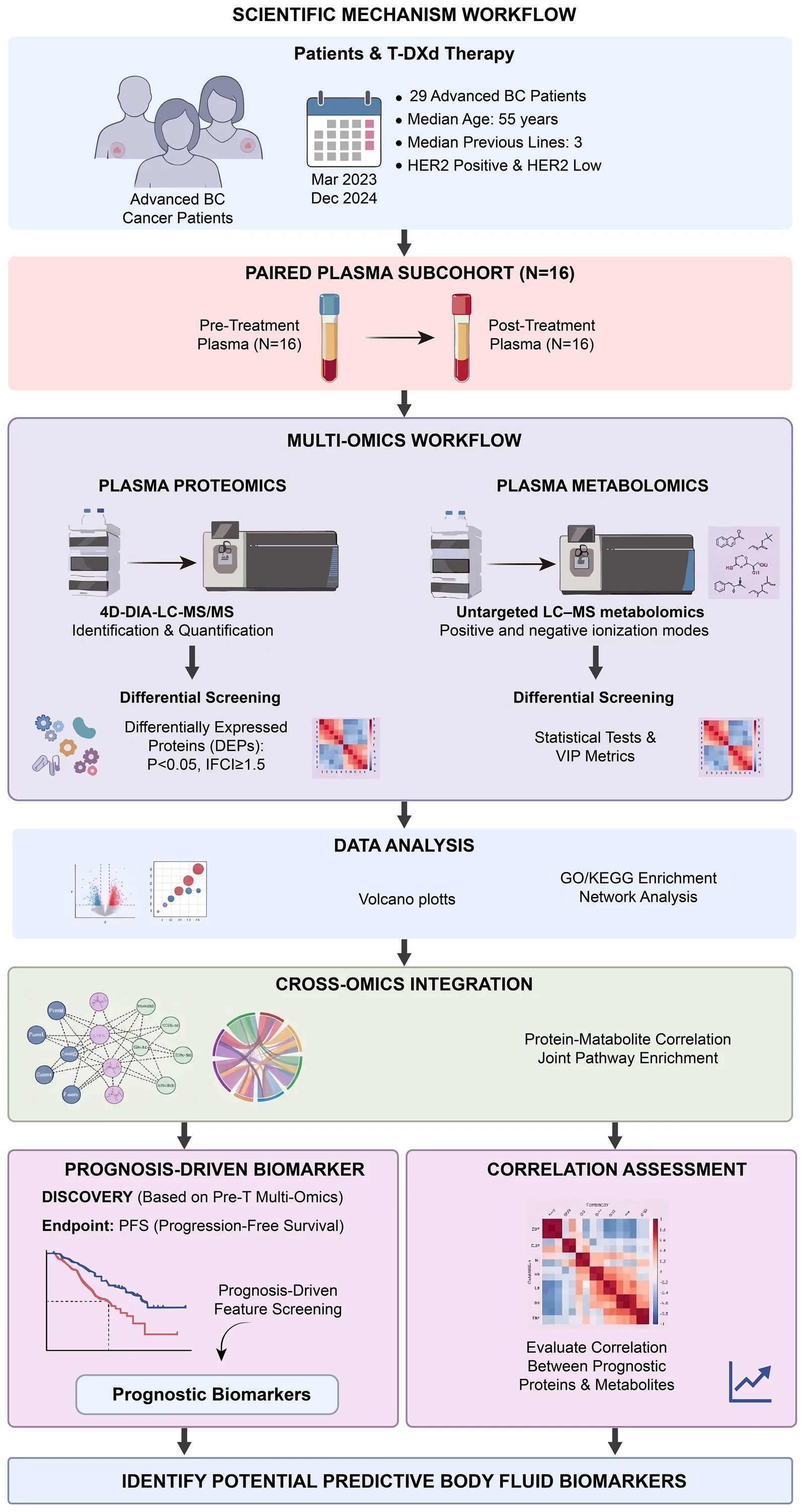
Workflow of this study.

### 2.2 Plasma proteomics

Plasma proteomics was performed on a 4D-DIA platform. Equal protein aliquots were denatured, reduced, alkylated, and digested with trypsin, followed by C18 solid-phase extraction. Across the sample set, 4,444 proteins corresponding to 53,811 peptides were identified. Proteins missing from more than 50% of samples were removed, and sporadic missing values were imputed using a k-nearest-neighbor procedure.

Differential protein abundance between paired pre- and post-treatment samples was assessed using a paired t test and fold-change analysis. Differentially expressed proteins were defined by a nominal, unadjusted P < 0.05 combined with FC > 1.5 or FC < 1/1.5. Under these criteria, 508 proteins were identified, including 136 upregulated and 372 downregulated proteins.

### 2.3 Plasma metabolomics

Untargeted plasma metabolomics was performed using UHPLC-high-resolution mass spectrometry. Plasma (100 uL) was mixed with 400 uL of pre-cooled methanol/acetonitrile (3:1), vortexed, incubated at 4 degrees C, centrifuged, dried under vacuum, and reconstituted in 50% methanol. Chromatographic separation used an ACQUITY UPLC HSS T3 column (1.8 um, 2.1 x 100 mm) at 40 degrees C and 0.3 mL/min. Samples were analyzed in positive- and negative-ion electrospray modes on a Thermo Q Exactive HF-X mass spectrometer using data-dependent acquisition over m/z 70-1050. Pooled QC samples were interspersed throughout the analytical sequence.

### 2.4 Multivariate statistics and functional enrichment

Raw metabolomics files were processed in Compound Discoverer for peak detection, alignment, extraction, retention-time correction, missing-value imputation, and normalization. Candidate annotations were assigned using MS/MS searches against mzCloud, ChemSpider, KEGG, HMDB, and the local mzVault library. Features were retained after mass-error and QC filtering (-5 to 5 ppm and CV <= 30%). PCA, PLS-DA, and OPLS-DA were used to summarize global data structure; permutation analysis was used to examine supervised-model robustness.

Differential metabolite features were defined by the conjunction of three criteria: paired t-test P < 0.05, OPLS-DA VIP > 1.0, and FC > 1.5 or FC < 1/1.5. As for the proteomic screen, the t-test P values were nominal and unadjusted; the 70 features are therefore reported as a discovery-stage set. VIP is a multivariate importance weight, not a significance test, and no P value attaches to it. Differential proteins and metabolite features were visualized using volcano plots, Z-score-normalized hierarchical heatmaps, correlation heatmaps, and representative violin plots. GO and KEGG over-representation analyses were applied to differential proteins, whereas differential metabolite features were mapped to KEGG pathways and displayed as bar, bubble, and chord plots.

### 2.5 Integrated protein–metabolite multi-omics analysis

Pearson correlation coefficients were calculated between treatment-associated differential proteins and metabolite features, and the strongest protein-metabolite relationships were summarized by clustered heatmaps. Differential proteins and metabolite features were also jointly mapped to KEGG pathways to identify shared functional contexts. These analyses were implemented in R and Python.

### 2.6 Prognosis-driven molecular selection and correlation analysis

PFS was defined as the interval from T-DXd initiation to documented disease progression or death. Univariable Cox proportional hazards regression was performed for standardized baseline protein and metabolite features. Features meeting the nominal threshold of P < 0.05 were considered for LASSO-Cox screening, with the penalty parameter selected by cross-validation. Kaplan-Meier curves were generated after median dichotomization of features selected for visualization, and groups were compared by the log-rank test. HRs are reported per standard-deviation increase with 95% CIs. Spearman correlation was used for the baseline candidate protein-metabolite matrix, and HER2 subgroups were compared using the Wilcoxon rank-sum test. Because of the limited sample size and high-dimensional screening, all PFS analyses were considered exploratory; nominal P values were not interpreted as independent validation.

## 3 Results

### 3.1 Patient clinical characteristics

A total of 16 patients with advanced breast cancer, nested within the 29-patient clinical cohort, were included for the paired multi-omics analysis; all molecular results below refer to this 16-patient cohort. Baseline clinical characteristics of the study cohort are summarized in **Table 1**. The cohort comprised 3 HER2-positive and 13 HER2-low patients. The median age was 55 years (range: 44–76 years), and the majority of patients were postmenopausal (14/16, 87.5%), with only 2 patients premenopausal (12.5%). Most patients had good performance status, and nearly all had undergone prior breast surgery. Five patients (31.2%) had baseline brain metastases, while 11 (68.8%) did not. The cohort was predominantly heavily pre-treated: 10 patients (62.5%) had received more than three prior lines of systemic therapy, while 6 (37.5%) had received three or fewer. Among the 16 patients, 3 achieved stable disease (SD) and 13 achieved partial response (PR), resulting in a disease control rate (DCR) of 100%. The median follow-up was 13.6 months, and disease progression occurred in 8 patients.

**Table 1:** Baseline characteristics of 16 patients with advanced breast cancer treated with T-DXd.

Clinicopathological characteristics	Total(N=16)	Percentage (%)
Age (years)		
Median (range)	55 (44-76)	
>50	11	68.80%
≤50	5	31.20%
Menopausal status		
Postmenopausal	14	87.50%
Premenopausal	2	12.50%
ECOG performance status.		
0-1	10	62.50%
2-4	6	37.50%
Prior breast cancer surgery		
Yes	14	87.50%
No	2	12.50%
Histological subtype		
ductal carcinoma	16	100.00%
HER2 status		
HER2-positive	3	18.80%
HER2-low	13	81.20%
Brain metastases		
Yes	5	31.20%
No	11	68.80%
Prior lines of systemic therapy		
>3 lines	10	62.50%
≤3 lines	6	37.50%

### 3.2 Metabolomic landscape of T-DXd treatment

QC samples clustered closely in the unsupervised analyses. PCA showed only partial group separation, with PC1 and PC2 explaining 13.3% and 10.4% of the variance, respectively (23.7% combined; **Supplementary Figure S2**). Supervised PLS-DA modeling achieved clear separation between the pre- and post-treatment groups, with high goodness-of-fit (R²Y) and predictive ability (Q²), and the permutation test reached statistical significance (P < 0.005; **Supplementary Figure S2**), confirming the robustness of the group discrimination. Based on paired statistical tests and multivariate analysis criteria, a total of 70 significantly differential metabolites were identified (after merging positive and negative ion modes and removing duplicates). As shown in the volcano plot (**Figure 2A**), the horizontal axis represents log2(FC) (post-treatment vs. pre-treatment) and the vertical axis represents –log10(p). Most metabolite points were distributed in the upper left (downregulated) or upper right (upregulated) region, with only a few in the non-significant area. Points with deeper color represent metabolites with greater contribution to group discrimination based on VIP values. For example, a VIP-high and significantly upregulated metabolite was identified as isoleucine, with a log2FC of approximately +0.8, indicating a ~1.74-fold increase after treatment (P < 0.01). Conversely, another important metabolite, tryptophan, was significantly decreased after treatment (log2FC ≈ –0.6, P < 0.05), suggesting that tryptophan and its downstream pathways may be suppressed. As shown in the hierarchical clustering heatmap (**Figure 2B**), Z-score normalized concentrations of all differential metabolites were subjected to bidirectional clustering, revealing obvious differences in metabolite abundance patterns between the pre-treatment group (mostly on the left) and post-treatment group (mostly on the right). Overall, the post-treatment group showed lower Z-scores (predominantly blue) for most differential metabolites, while the pre-treatment group showed relatively higher scores (predominantly red) for clusters including various amino acids and energy metabolites, indicating that these molecules decreased after treatment. In contrast, a small cluster of metabolites was significantly elevated in the post-treatment group, including some lipid molecules and metabolic regulators. The specific changes of each differential metabolite were also displayed by violin plots (**Figure 2D**).

**Figure 2:**
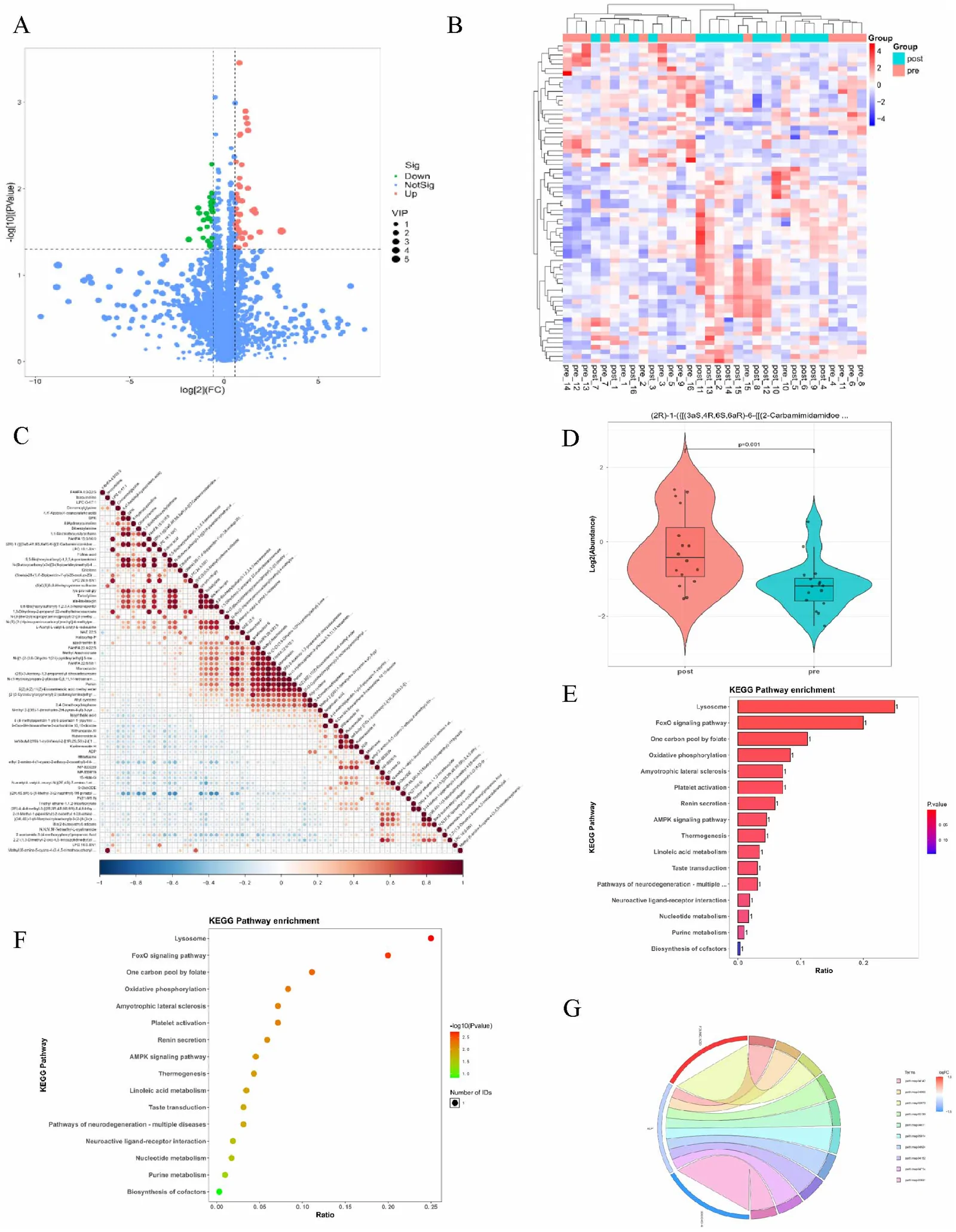
Treatment-associated metabolomic features. **(A)** Volcano plot of differential metabolite features. **(B)** Hierarchical heatmap of differential features. **(C)** Correlation heatmap. **(D)** Violin plot of a representative differential feature. **(E)** KEGG pathway bar plot. **(F)** KEGG pathway bubble plot. **(G)** Metabolite-pathway chord plot.

To further decompose the variance structure, orthogonal partial least squares discriminant analysis (OPLS-DA) was subsequently applied to isolate and remove intra-group orthogonal variation unrelated to treatment group assignment. Following removal of the orthogonal component, the residual inter-group differences were attributable to a discrete subset of treatment-responsive metabolite features rather than non-specific spectral variation. Permutation testing of the OPLS-DA model confirmed statistical robustness against overfitting (P < 0.005; **Supplementary Figure S2**), validating the observed metabolomic perturbations as genuine treatment-associated signals rather than model artifacts.

Pathway enrichment analysis of the identified differential metabolites showed that amino acid metabolism and lipid metabolism-related pathways were significantly enriched among the T-DXd treatment-associated differences. In the KEGG pathway enrichment analysis, the most significantly enriched pathways included “Lysosome,” “FoxO signaling pathway,” “One carbon pool by folate,” and several other metabolic routes. These pathways contained multiple members among the differential metabolites, and most showed consistent directions of change. As shown in the KEGG enrichment bar chart (**Figure 2E**), the counts of upregulated (red) and downregulated (blue) metabolites in each pathway are clearly displayed. The enrichment bubble plot (**Figure 2F**) and chord diagram (**Figure 2G**) further illustrate the differential pathways.

Note: In panel A, the x-axis represents log2FC (post-treatment vs. pre-treatment), and the y-axis represents -log10(P value). Red, green, and blue denote upregulated, downregulated, and non-significant features, respectively. Heatmaps show standardized relative abundance or correlation coefficients. Pathway plots summarize the KEGG mapping of the 70 differential metabolite features.

### 3.3 Proteomic landscape of T-DXd treatment

Plasma proteomics identified 4,444 proteins. Paired comparison yielded 508 differential proteins after T-DXd treatment, including 136 upregulated and 372 downregulated proteins (**Figure 3A**). The heatmap showed heterogeneous sample-level patterns and did not establish complete unsupervised separation of the treatment groups (**Figure 3B**); a representative differential protein is shown in **Figure 3C**. GO enrichment was dominated by cytoplasmic translation, translation, ribosomal-subunit biogenesis, rRNA processing, membrane and cytosolic-ribosome components, and RNA- or cytoskeleton-related molecular functions (**Figure 3D**). KEGG analysis similarly highlighted ribosome, focal adhesion, gap junction, calcium signaling, cGMP-PKG signaling, leukocyte transendothelial migration, and platelet activation (**Figures 3E, F**). Disease-named KEGG terms were interpreted as shared molecular modules rather than evidence of the corresponding clinical conditions. The subcellular distribution of differential proteins is summarized in **Figure 3G**. The STRING network identified a densely connected hub subnetwork dominated by complement and coagulation proteins, including C2, C4, Factor B, plasminogen, and antithrombin (**Figure 3H**).

**Figure 3:**
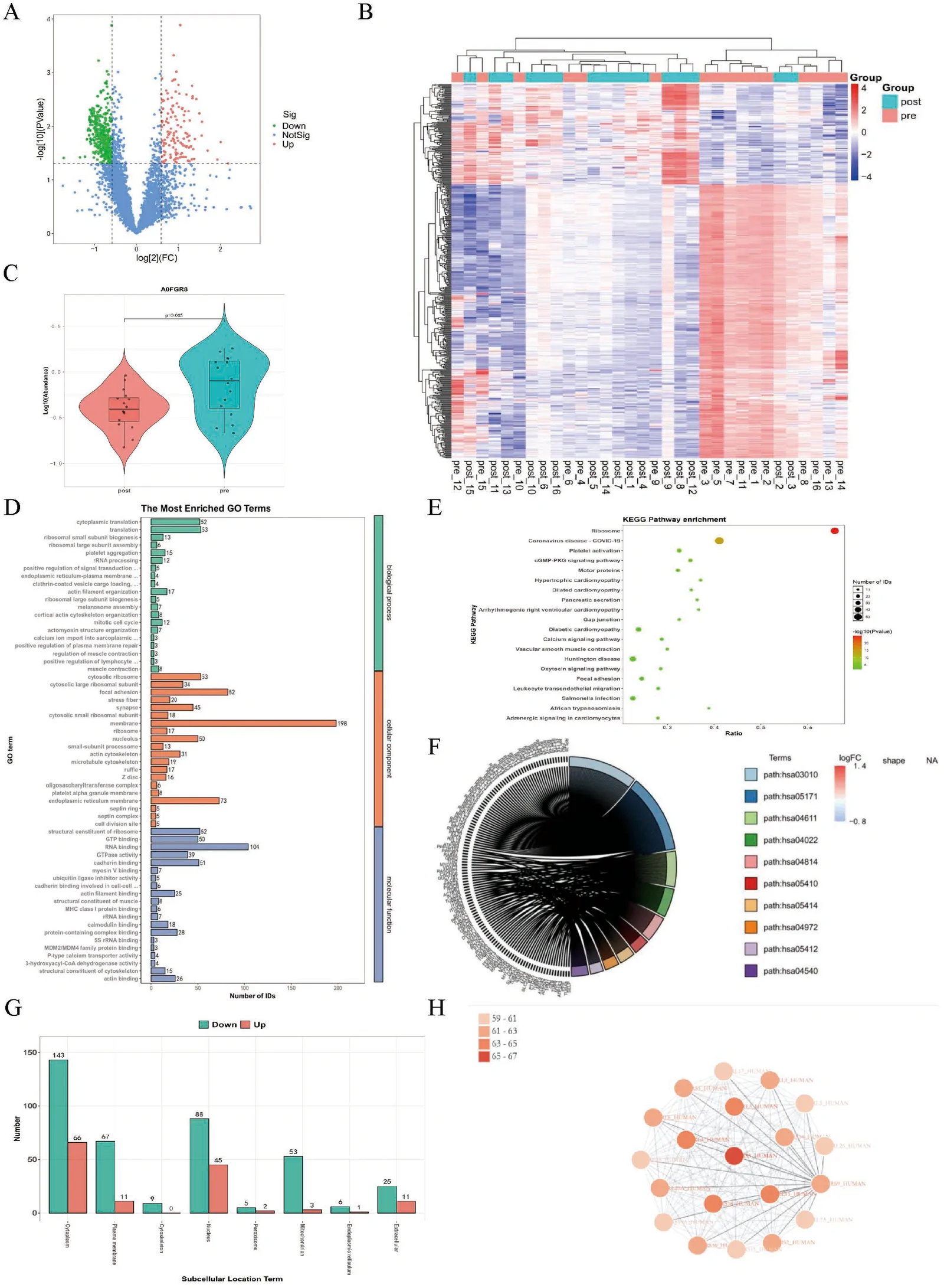
Analysis of treatment-associated differential proteins. **(A)** Volcano plot. **(B)** Hierarchical heatmap. **(C)** Violin plot of a representative differential protein. **(D)** GO enrichment bar plot. **(E)** KEGG enrichment bubble plot. **(F)** KEGG chord plot. **(G)** Subcellular localization distribution. **(H)** STRING PPI hub network. The PPI network was generated using Homo sapiens identifiers, and node color represents degree.

Among the most prominently downregulated DEPs, the canonical acute-phase reactants C-reactive protein (CRP) and serum amyloid A (SAA) exhibited log2 fold changes of approximately −1 to −2 (P < 0.01), which is compatible with attenuation of systemic inflammatory activity during treatment. However, we note that inflammatory activity remains an interpretation of a plasma abundance change rather than a demonstrated reduction in inflammation. In contrast, several DEPs demonstrated post-treatment upregulation, including complement component C4 (log2FC ≈ +0.6, P < 0.05) and apolipoprotein A-I (ApoA1). Immune reconstitution and altered high-density lipoprotein-mediated lipid transport are two candidate explanations for this pattern. Individual DEPs were further evaluated by paired violin plots and receiver operating characteristic (ROC) analysis; single-protein AUC values were predominantly in the range of 0.60–0.70, underscoring the inherent limitations of individual biomarker performance and supporting the necessity of multi-marker integration to achieve adequate discriminative capacity.

Betweenness- and degree-centrality analyses of the STRING PPI network identified albumin (ALB) as the highest-degree hub node, bridging lipoprotein metabolism, complement activation, and coagulation functional modules, consistent with its established role as a pleiotropic plasma carrier protein. The fibrinogen subunit complex (FGA, FGB, and FGG) constituted a tightly interconnected hub cluster within the coagulation module and was uniformly and markedly downregulated, indicating a coordinated reduction in coagulation network activity. Complement C3, positioned centrally within the complement cascade sub-network, was likewise substantially decreased post-treatment. These high-connectivity hub proteins are predicted to exert disproportionate regulatory influence across multiple downstream biological networks and may therefore represent principal modulatory nodes in the systemic proteomic response to T-DXd therapy.

To characterize the biological origins of the differential proteome, the 508 DEPs were annotated against the Human Protein Atlas (HPA) plasma proteome reference. Over 60% of DEPs were classified as known or predicted secretory proteins, the majority of which are liver-synthesized classical plasma constituents including complement factors, coagulation cascade components, and apolipoproteins. The predominant post-treatment decrease in this hepatic-derived protein fraction likely reflects a combination of reduced acute-phase synthesis secondary to diminished tumor burden and attenuated systemic inflammation. Approximately 15% of DEPs were annotated as blood-cell-derived or membrane-associated proteins, whose downregulation is consistent with a transition toward a less activated peripheral immune compartment. A small subset (approximately 5%) comprised tissue-enriched proteins; of note, cardiac troponin T (cTnT) was elevated in a minority of patients following treatment, a pattern that warrants caution rather than a definitive interpretation of subclinical myocardial stress. This finding should not be overinterpreted as evidence of subclinical myocardial injury or treatment-related cardiotoxicity without appropriate clinical correlation.

### 3.4 Integrated multi-omics analysis

To examine coordinated treatment-associated changes, we calculated correlations between differential proteins and metabolite features. The heatmap revealed structured positive and negative modules rather than uniformly near-perfect correlations (**Figure 4A**). PRG2, LEG3, RL14, RL21, and RL17 showed positive correlations with several metabolite features, whereas PDIA5 and RSU1 showed negative correlations with a subset of features. These associations describe co-variation and do not establish direct biochemical regulation.

**Figure 4:**
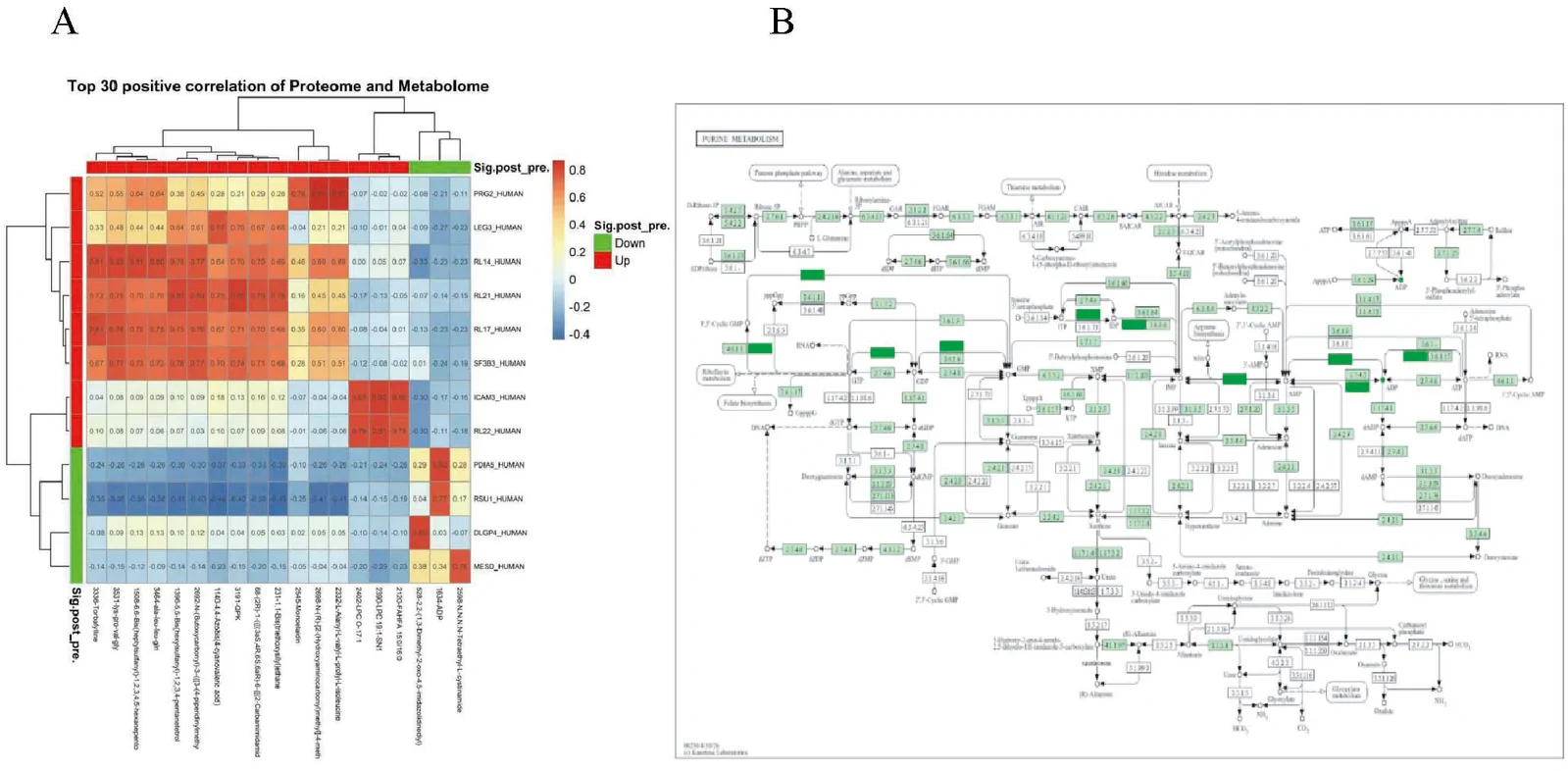
Cross-omics integration. **(A)** Clustered heatmap of top correlated protein-metabolite pairs. **(B)** Joint mapping of differential proteins and metabolite features to the KEGG purine-metabolism pathway. Correlation coefficients indicate co-variation and should not be interpreted as causal interactions.

Joint KEGG mapping identified several pathways with concordant changes at both protein and metabolite levels. In branched-chain amino acid metabolism, key enzymes BCAT1 and BCKDHB were upregulated at the protein level, consistent with metabolite shifts and supporting coordinated metabolic axis remodeling. Purine metabolism exhibited suppression-aligned patterns, with both metabolite (e.g., hypoxanthine) and enzyme (e.g., ADA) level reductions suggesting proliferation-associated nucleotide metabolic inhibition. These cross-omics signatures collectively delineate a multi-module remodeling landscape (**Figure 4B**).The pathway map localizes the detected features within the purine network and is presented as an exploratory integration result.

An additional cross-omics convergence was identified in the arachidonic acid metabolic pathway. Post-treatment plasma levels of arachidonic acid and several of its downstream eicosanoid derivatives were elevated, whereas cyclooxygenase-2 (COX-2) protein abundance showed a numerically lower post-treatment mean, but this difference did not meet our differential-expression criteria. This pattern raises the possibility that T-DXd therapy attenuates downstream prostaglandin biosynthesis while permitting accumulation of the upstream arachidonic acid precursor pool, but this remains speculative.

### 3.5 Prognostic molecular features derived from pre-treatment multi-omics data

Exploratory univariable Cox screening of baseline profiles yielded 57 protein(**Supplementary Table S1**) and 113 metabolite features at nominal P < 0.05.Among the 508 treatment-associated DEPs, univariable Cox analysis identified 8 proteins significantly associated with PFS (**Table 2**). In parallel, LASSO-Cox screening across all 4,444 baseline proteins selected 6 protein candidate(CLCN4, RCAN2, ALDH3B1, SLC16A1, PABPC1, and CEMIP) and four metabolite candidates (**Supplementary Figures S3, S5**). Their univariable HR estimates are reported in **Tables 3**, **2** and correspond directly to the features displayed in **Figures 5**, **6**. Because only 16 baseline profiles were available and no external validation cohort was used, these results are hypothesis-generating rather than validated prognostic signatures.

**Table 2:** Hazard ratio analysis of differential proteins for PFS.

Gene	HR	95%CI low	95%CI high	*P*
IGHV1-18	2.209	1.067	4.574	0.033
INPP5B	2.222	1.031	4.786	0.041
ALDH3B1	2.405	1.065	5.43	0.035
SLC16A1	3.159	1.145	8.717	0.026
RPL15	2.813	1.092	7.249	0.032
ZNF384	2.378	1	5.651	0.05
EXOC6B	2.278	1.072	4.841	0.032
SH3BP1	3.223	1.306	7.957	0.011

HR, hazard ratio; CI, confidence interval; SD, standard deviation. HRs were estimated by univariable Cox proportional hazards regression per 1-SD increase in log-transformed baseline abundance.

**Table 3:** Hazard ratio analysis of differential metabolites for PFS.

Differential metabolites	HR	95%CI low	95%CI high	*P*
6,6-Bis(heptylsulfanyl)-1,2,3,4,5-hexanepentol	2.635	1.216	5.708	0.014
Dibenzylamine	2.062	1.007	4.222	0.048
Isoquinoline	0.406	0.17	0.971	0.043
Methyl [6-amino-5-cyano-4-(3,4,5-trimethoxyphenyl) -2,4-dihydropyrano[2,3-c]pyrazol-3-yl]acetate	0.176	0.031	0.991	0.049
ala-leu-leu-gln	2.675	1.232	5.804	0.013

HR, hazard ratio; CI, confidence interval; SD, standard deviation. HRs were estimated by univariable Cox proportional hazards regression per 1-SD increase in log-transformed baseline abundance.

**Figure 5:**
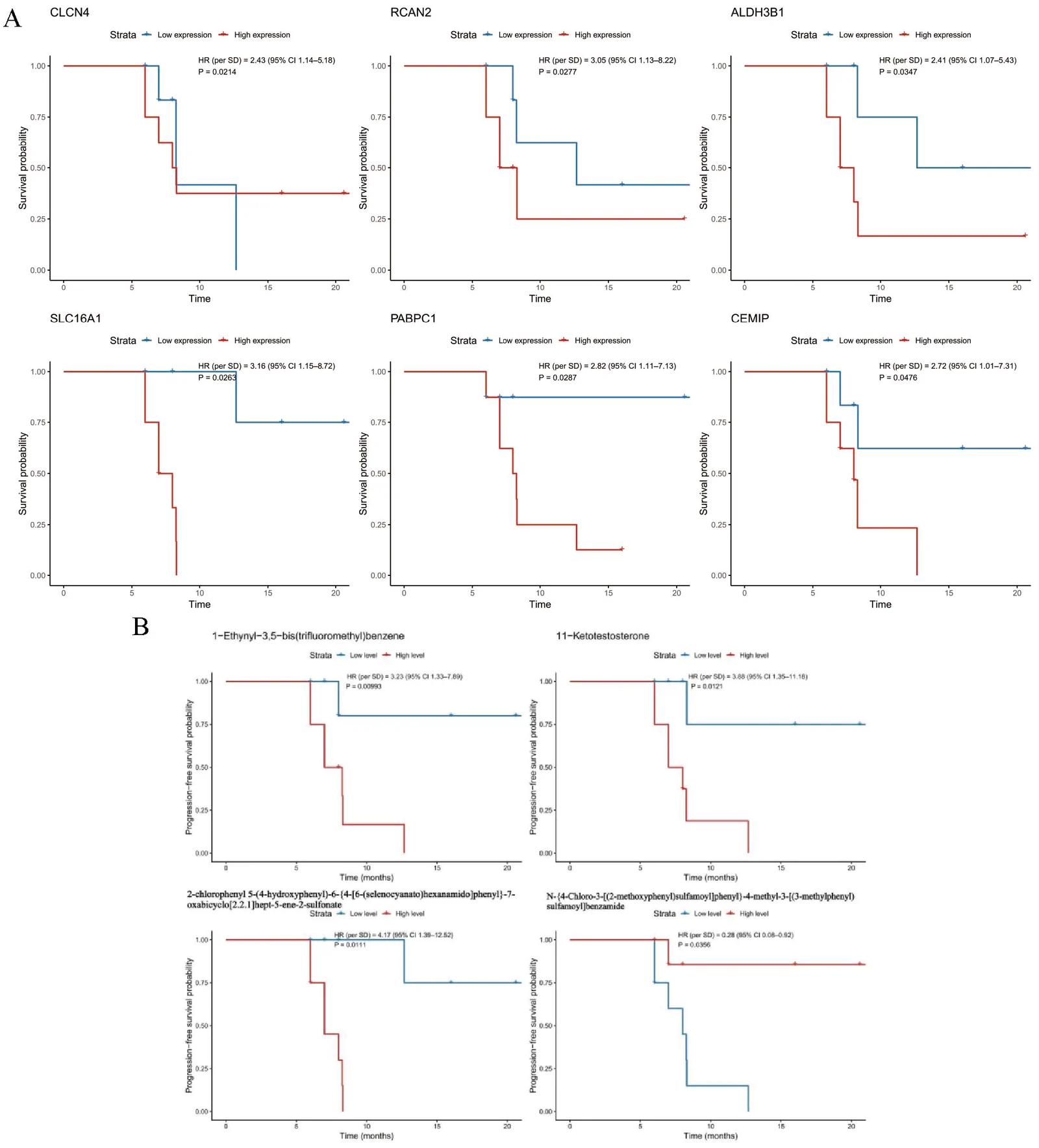
Exploratory PFS visualization of baseline candidate features. **(A)** Kaplan-Meier curves for six protein candidates based on pre-treatment baseline levels. **(B)** Kaplan-Meier curves for four metabolite candidates based on pre-treatment baseline levels. Patients were dichotomized at the median. Blue and red lines denote lower and higher baseline abundance, respectively; tick marks denote censoring. HRs and 95% CIs are from univariable Cox models.

**Figure 6:**
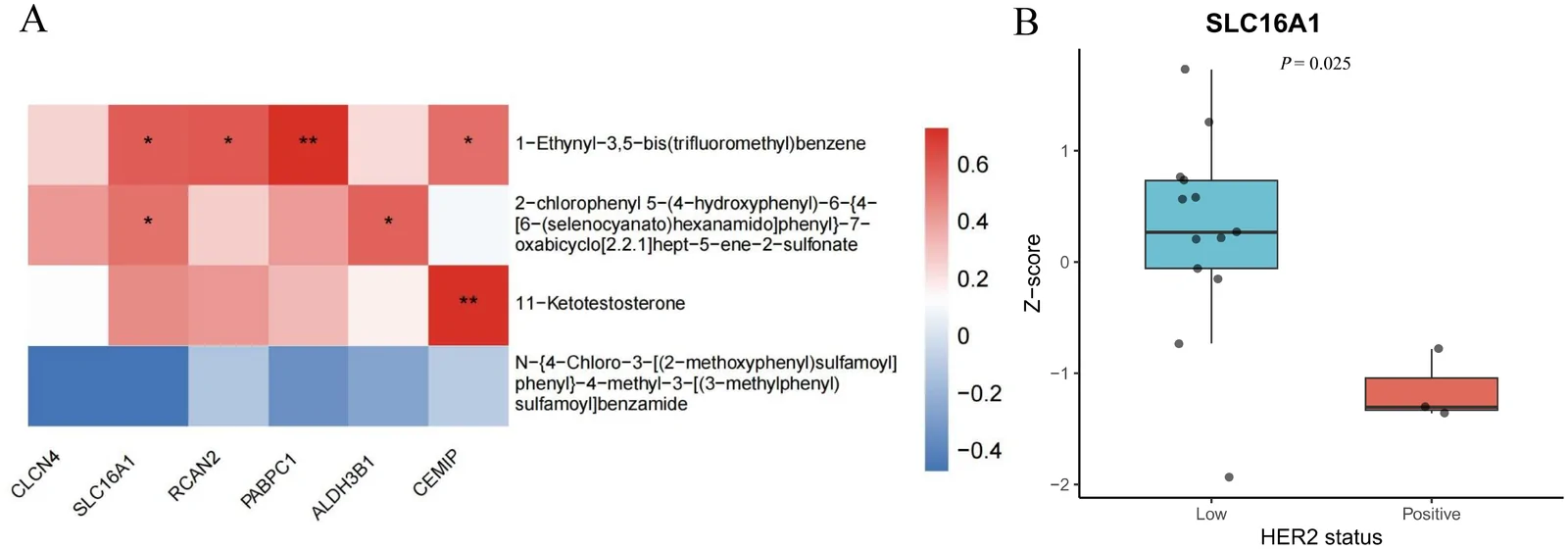
Exploratory correlation and subgroup analyses of baseline candidates. **(A)** Spearman correlation heatmap for six protein and four metabolite candidates. **(B)** Baseline SLC16A1 abundance according to HER2 status. In panel **(A)**, asterisks indicate statistical significance (*P < 0.05, **P < 0.01); correlation coefficients range from −1 to +1.

Spearman correlation analysis of the six protein and four metabolite candidates revealed several nominally significant pairwise associations (**Figure 6A**). SLC16A1, RCAN2, PABPC1, and CEMIP each demonstrated positive correlations with the metabolite feature 1-ethynyl-3,5-bis(trifluoromethyl)benzene (Spearman ρ ≈ 0.54–0.72; P < 0.05), with the PABPC1–metabolite pair exhibiting the strongest association (ρ ≈ 0.72, P < 0.01). SLC16A1 and ALDH3B1 were additionally and positively correlated with a second candidate metabolite feature (P < 0.05). CEMIP showed a strong positive association with 11-ketotestosterone (ρ ≈ 0.73, P < 0.01). The observed correlation structure—in which prognostic proteins implicated in membrane transport, cellular stress response, and metabolic regulation co-vary with lipid-related and steroid-like metabolite features—suggests that pre-treatment proteomic and metabolomic states may reflect shared or coupled biological processes relevant to disease progression and treatment sensitivity. In the exploratory HER2 subgroup comparison based on pre-treatment plasma profiles, only SLC16A1 showed a nominally significant between-group difference, with higher abundance in the HER2-low subgroup relative to the HER2-positive subgroup (P = 0.025, Wilcoxon rank-sum test; **Figure 6B**). The remaining prognostic candidate proteins and metabolites did not demonstrate statistically significant subgroup differences, indicating that the identified multi-omics prognostic signature does not, in aggregate, closely parallel HER2 expression status. These observations suggest that HER2 phenotype may be associated with individual circulating molecular features rather than serving as a predominant determinant of the overall baseline multi-omics prognostic landscape. Given the small subgroup sizes (HER2-positive: n = 3; HER2-low: n = 13), these findings are hypothesis-generating and require confirmation in larger, prospectively collected cohorts with stratified HER2 subgroup representation.

Note: Color depth represents the Spearman correlation coefficient. *P < 0.05 and **P < 0.01. The box plot shows SLC16A1 Z-scores; the center line denotes the median, and points denote individual patients.

## 4 Discussion

This paired plasma multi-omics study identified treatment-associated changes across the proteome and metabolome during T-DXd therapy. Our analyses support a broad systemic remodeling of the plasma proteome, characterized by coordinated downregulation of complement, coagulation, and acute-phase inflammatory proteins, alongside upregulation of certain lipid transport and metabolic regulatory proteins, as well as remodeling of translation/ribosome-related and cytoskeletal/adhesion pathways. These changes, together with heterogeneous metabolic features and structured cross-omics co-variation, depict a multi-layered molecular response involving both tumor-intrinsic and host systemic compartments. Baseline Cox and LASSO-Cox analyses generated exploratory PFS-associated candidates, but the study was not designed to establish a validated predictive biomarker model.

Although complement and coagulation proteins constituted a biologically prominent fraction of the downregulated DEPs, GO and KEGG enrichment of all 508 DEPs was primarily driven by translation- and ribosome-related processes. Instead, GO and KEGG results were centered on translation, ribosomal organization, cytoskeletal and membrane components, focal adhesion, intercellular signaling, and platelet-related processes. These results indicate broad remodeling of circulating protein patterns. The prominent downregulation of acute-phase proteins (e.g., CRP, SAA) is consistent with reduced tumor burden and systemic inflammation following T-DXd therapy (13, 14), while the upregulation of translation/ribosome-related proteins may reflect adaptive stress responses in the tumor or host compartment (15). Future studies incorporating paired tumor biopsies could help resolve the tissue origins of these signals.

The metabolomics analysis identified 70 differential features, with 43 increasing and 27 decreasing after treatment. Unsupervised PCA and hierarchical clustering showed substantial overlap between pre- and post-treatment samples, and the supervised PLS-DA model showed clear group separation with permutation testing reaching statistical significance (P < 0.005). The top-ranked KEGG terms included lysosome, FoxO signaling, one-carbon metabolism, oxidative phosphorylation, and platelet activation. Although some pathway terms were supported by a limited number of features, they provide initial clues to the metabolic pathways affected by T-DXd (16) and highlight candidates for targeted follow-up studies (17).The elevation of branched-chain amino acids, particularly isoleucine, may reflect reduced tumor uptake following T-DXd-induced proliferative arrest (18), providing a plausible explanation for the observed increase in circulating BCAA levels.

The cross-omics heatmap showed biologically structured positive and negative co-variation, and joint mapping highlighted purine metabolism. These results describe co-variation among circulating features (e.g., BCAT1/BCKDHB with branched-chain amino acids, NAMPT with tryptophan-niacin axis). Co-variation in plasma, however, is not evidence of enzyme-substrate coupling: pathway relationships are database-derived, metabolic flux was not measured, and tissue origins are unknown. Metabolic adaptation to T-DXd-induced tumor cell stress is a plausible interpretation. Together with the purine metabolism suppression pattern (hypoxanthine and ADA reduction), these findings generate testable hypotheses regarding enzyme-substrate coordination and potential resistance mechanisms. The downregulation of NAMPT in the tryptophan-niacin axis, coupled with kynurenine elevation, is consistent with altered IDO activity and may have implications for antitumor immunity (19). Moreover, T-DXd-induced tumor cell death may trigger immunogenic cell death (ICD), activating STING/TLR4 pathways and enhancing T-cell-mediated antitumor immunity (20, 21). The observed metabolite shifts in pathways related to ABC transporter function, alongside changes in polyamines such as spermidine whose transmembrane transport is ABC transporter-dependent, are consistent with previously reported ABC transporter-mediated payload efflux as a resistance mechanism (22, 23). Arginine and IDO axis modulation could be targeted by combination therapies to enhance T-DXd efficacy (24). Functional experiments and longitudinal sampling at progression are warranted to confirm these relationships.

The resulting separation of Kaplan-Meier curves is hypothesis-generating and warrants further validation in larger, independent cohorts to evaluate its potential clinical relevance. Higher pre-treatment isoleucine levels in patients who responded more favorably (25), and extremely elevated baseline inflammatory proteins (e.g., CRP) associated with inferior long-term outcomes (26), suggest that pre-treatment multi-omics profiling could facilitate patient stratification. The metabolic status of tumor and host, as well as cellular transport capacity, are closely linked to intracellular payload exposure from ADC drugs, thereby influencing treatment sensitivity and resistance risk (27–29). Pre-treatment metabolic phenotypes can reflect tumor-host metabolic coupling and have demonstrated predictive and prognostic value across various antitumor therapeutic contexts (30, 31). The coordinated protein-metabolite co-variation observed here is particularly prominent in amino acid metabolism, lipid transport, and transmembrane transport pathways—consistent with the biological mechanism by which ADC drugs depend on the intracellular metabolic environment and transport machinery to exert cytotoxic effects (32).It should be noted that some differential metabolite features were annotated based on top spectral-library matches and await confirmation by authentic standards; therefore, these entities are referred to as metabolite features or candidates. The same caution applies to pathway mapping that depends on those annotations.

We recognize that the clinical utility of these findings remains hypothetical. Early response monitoring is the most plausible: the treatment-associated changes were detectable after only two cycles, and a plasma assay measuring a small panel of consistently moving proteins could in principle indicate early systemic response with only a blood draw. However, our study did not test whether these changes track radiological response and establishing that link is a prerequisite. Meanwhile, before any application is possible, candidate features must replicate in an independent cohort, metabolite annotations must be confirmed against authentic standards, and clinical utility must be demonstrated against routine restaging and standard laboratory parameters.

This study has several limitations. The cohort was retrospective, only 16 patients had paired multi-omics profiles, the number of screened molecular features was large relative to the number of PFS events. Multiple-testing correction was not applied to the feature-level differential analyses. Therefore, individual proteins and metabolites should be considered exploratory candidates. No independent validation cohort was available. Brain metastasis status was recorded as a potential source of plasma profile heterogeneity, but the small subgroup size precluded meaningful comparative analysis. In addition, associations with PFS are prognostic within this treated cohort; distinguishing predictive from prognostic effects would require a comparator arm not receiving T-DXd.

## 5 Conclusions

In summary, paired plasma proteomics and metabolomics revealed treatment-associated molecular remodeling during T-DXd therapy, with functional results emphasizing translation/ribosome-related remodeling as the dominant proteomic signature, accompanied by downregulation of complement/coagulation and acute-phase pathways, alongside lipid transport/metabolism modulation, as well as amino acid, nucleotide, and energy metabolic reprogramming. Exploratory baseline analyses generated six protein and four metabolite candidates associated with PFS visualization.

## Data Availability

The data analyzed in this study is subject to the following licenses/restrictions: The datasets generated during the current study are not publicly available to protect patient privacy but are available from the corresponding author on reasonable request. Requests to access these datasets should be directed to sangdie0123@126.com.
