# Supplementary material for: Plasma multi-omics reveals molecular remodeling and prognostic biomarkers in advanced breast cancer treated with trastuzumab deruxtecan
Source: Front Oncol. 2026 Sep 18;16:1973097. doi: 10.3389/fonc.2026.1973097 (PMC13630672; doi:10.3389/fonc.2026.1973097)
Supplement: Supplementary file 1 [file DataSheet1.docx]

**Supplement Material**

Figure S1. Flowchart of patients selection

Figure S2. Quality control and clustering analysis of metabolomic samples.A)Hierarchical clustering analysis of metabolomic samples (including QC).B) PCA score plot of metabolomic samples (including QC).C) PLS-DA score plot of metabolomic samples (including QC).D) PLS-DA model and permutation test

Figure S3. LASSO-Cox regression analysis of prognostic models.A)Coefficient path plot of LASSO-Cox model based on pre-treatment proteomicsB) Cross-validation curve of LASSO-Cox model based on pre-treatment proteomicsC) Coefficient path plot of LASSO-Cox model based on pre-treatment metabolomicsD) Cross-validation curve of LASSO-Cox model based on pre-treatment metabolomics

Table S1. **Univariable Cox regression analysis of pre-treatment protein expression with PFS.**

| **Gene** | **HR** | **95%CI low** | **95%CI high** | ***P*** |
| --- | --- | --- | --- | --- |
| SH3BP1 | 3.223 | 1.306 | 7.957 | 0.011 |
| PIK3R6 | 3.241 | 1.302 | 8.068 | 0.012 |
| CA2 | 2.566 | 1.211 | 5.44 | 0.014 |
| MXRA5 | 2.455 | 1.189 | 5.067 | 0.015 |
| COL5A3 | 3.159 | 1.246 | 8.007 | 0.015 |
| TFAM | 2.438 | 1.171 | 5.077 | 0.017 |
| SQSTM1 | 2.575 | 1.175 | 5.642 | 0.018 |
| CLCN4 | 2.431 | 1.141 | 5.183 | 0.021 |
| ARFGAP2 | 2.965 | 1.166 | 7.539 | 0.022 |
| PABPC4 | 2.68 | 1.143 | 6.283 | 0.023 |
| TMEM165 | 2.996 | 1.156 | 7.765 | 0.024 |
| GFPT1 | 2.424 | 1.121 | 5.241 | 0.024 |
| SLC25A46 | 2.628 | 1.13 | 6.112 | 0.025 |
| DNMT1 | 2.574 | 1.122 | 5.904 | 0.026 |
| ALG5 | 2.229 | 1.101 | 4.515 | 0.026 |
| PLAT | 2.414 | 1.11 | 5.248 | 0.026 |
| SLC16A1 | 3.159 | 1.145 | 8.717 | 0.026 |
| ADGRE3 | 2.361 | 1.105 | 5.044 | 0.027 |
| ATP6AP1 | 2.496 | 1.106 | 5.63 | 0.028 |
| RCAN2 | 3.048 | 1.13 | 8.22 | 0.028 |
| PABPC1 | 2.818 | 1.114 | 7.13 | 0.029 |
| DENND4A | 2.976 | 1.113 | 7.956 | 0.03 |
| NAA35 | 2.458 | 1.082 | 5.585 | 0.032 |
| EXOC6B | 2.278 | 1.072 | 4.841 | 0.032 |
| RPL15 | 2.813 | 1.092 | 7.249 | 0.032 |
| MEAK7 | 2.367 | 1.075 | 5.215 | 0.032 |
| FNTB | 2.243 | 1.069 | 4.704 | 0.033 |
| IGHV1-18 | 2.209 | 1.067 | 4.574 | 0.033 |
| ALDH3B1 | 2.405 | 1.065 | 5.43 | 0.035 |
| BLMH | 2.26 | 1.054 | 4.847 | 0.036 |
| IGF2 | 2.345 | 1.049 | 5.239 | 0.038 |
| CA1 | 2.15 | 1.04 | 4.443 | 0.039 |
| ELMO2 | 2.246 | 1.036 | 4.871 | 0.04 |
| INPP5B | 2.222 | 1.031 | 4.786 | 0.041 |
| IGHV1-69 | 2.097 | 1.029 | 4.275 | 0.042 |
| BRI3BP | 2.101 | 1.029 | 4.289 | 0.042 |
| ARRB2 | 2.091 | 1.028 | 4.252 | 0.042 |
| RNASEH2B | 2.197 | 1.028 | 4.696 | 0.042 |
| NUDT5 | 2.151 | 1.022 | 4.527 | 0.044 |
| VCAN | 2.34 | 1.02 | 5.369 | 0.045 |
| CD69 | 2.139 | 1.017 | 4.501 | 0.045 |
| SIKE1 | 2.094 | 1.015 | 4.319 | 0.045 |
| DBT | 2.357 | 1.018 | 5.46 | 0.045 |
| OPLAH | 2.445 | 1.017 | 5.876 | 0.046 |
| CRIP2 | 1.989 | 1.013 | 3.903 | 0.046 |
| FERMT2 | 2.308 | 1.015 | 5.247 | 0.046 |
| CORO7 | 2.107 | 1.012 | 4.39 | 0.046 |
| PIP4K2C | 2.166 | 1.008 | 4.654 | 0.048 |
| CEMIP | 2.719 | 1.011 | 7.314 | 0.048 |
| MPI | 2.266 | 1.008 | 5.096 | 0.048 |
| SLC9A1 | 2.174 | 1.004 | 4.706 | 0.049 |
| EPRS1 | 1.846 | 1.002 | 3.399 | 0.049 |
| PLCB4 | 2.544 | 1.003 | 6.455 | 0.049 |
| GSS | 2.074 | 1.001 | 4.294 | 0.049 |
| HMOX1 | 2.558 | 1.001 | 6.532 | 0.049 |
| LAMA4 | 2.306 | 1 | 5.318 | 0.049 |
| ZNF384 | 2.378 | 1 | 5.651 | 0.049 |
